# Ubiquitin-Dependent Regulation of the Mammalian Hippo Pathway: Therapeutic Implications for Cancer

**DOI:** 10.3390/cancers10040121

**Published:** 2018-04-17

**Authors:** Thanh Hung Nguyen, Jan-Michael Kugler

**Affiliations:** Institute of Cellular and Molecular Medicine, University of Copenhagen, Blegdamsvej 3, 2200 Copenhagen, Denmark

**Keywords:** Hippo pathway, ubiquitination, ubiquitin E3 ligase, deubiquitination, deubiquitinating enzymes, oncogenic transformation

## Abstract

The Hippo pathway serves as a key barrier for oncogenic transformation. It acts by limiting the activity of the proto-oncogenes YAP and TAZ. Reduced Hippo signaling and elevated YAP/TAZ activities are frequently observed in various types of tumors. Emerging evidence suggests that the ubiquitin system plays an important role in regulating Hippo pathway activity. Deregulation of ubiquitin ligases and of deubiquitinating enzymes has been implicated in increased YAP/TAZ activity in cancer. In this article, we review recent insights into the ubiquitin-mediated regulation of the mammalian Hippo pathway, its deregulation in cancer, and possibilities for targeting the Hippo pathway through the ubiquitin system.

## 1. Introduction

The Hippo pathway is an important signaling network controlling cell growth and organ size during development. Hippo signaling is conserved between species as diverse as Drosophila and as humans (reviewed in [1]). The core mammalian Hippo pathway comprises a cassette of the upstream “mammalian Ste20-like kinases 1 and 2” (MST1/2) and the downstream “large tumor suppressors 1 and 2” (LATS1/2). The activity of these core Hippo kinases is influenced by regulatory proteins, Sav1 and RASSF proteins (acting on MST1/2), and MOB1 (acting on LATS1/2), and also by upstream signals, including G-protein coupled receptor signaling, KIBRA and NF2 [2,3,4]. MST kinases phosphorylate the LATS kinases, which in turn phosphorylate the proto-oncogenes YAP and TAZ at several serine residues leading to their inactivation through cytoplasmic retention and subsequent destabilization [5]. Unphosphorylated by Hippo kinases, YAP and TAZ translocate to the nucleus, where they act as transcriptional co-activators by binding to several transcription factors, including TEA domain family proteins (TEADs), β-catenin, and members of the RUNX family. Thereby, they promote transcription of target genes that stimulate cell growth and proliferation and inhibit apoptosis (reviewed in [6]).

Recent studies suggest that the Hippo pathway serves as a key barrier against oncogenic transformation by repressing the activity of YAP and TAZ [7,8,9,10]. Inhibition of Hippo pathway activity or expression of active YAP is required for efficient transformation in genetically controlled transformation models of human cells [11]. Increased nuclear activity of YAP and TAZ, as a result of their overexpression or suppressed activity of upstream Hippo kinases and regulators, is frequently observed in human cancer [12,13,14]. Loss-of-function mutations in NF2, a promoter of Hippo signaling acting on YAP [15], are often found in various types of human tumors [7]. In mouse models, YAP overexpression led to overgrowth of the liver, and this phenotype was reverted when ectopic YAP expression was terminated [16,17]. Similarly, inhibition of the core Hippo components MST1 and MST2, their regulators SAV1 and RASSF proteins, or upstream regulators such as NF2 resulted in overgrowth of the mouse liver and eventually in hepatocellular carcinoma [18,19,20]. Importantly, these phenotypes were suppressed by YAP depletion, substantiating the essential role of the Hippo-YAP signaling axis in oncogenic transformation. These data establish the Hippo pathway as an important suppressor of oncogenic transformation and indicate its relevance for human cancer.

**Figure 1:**
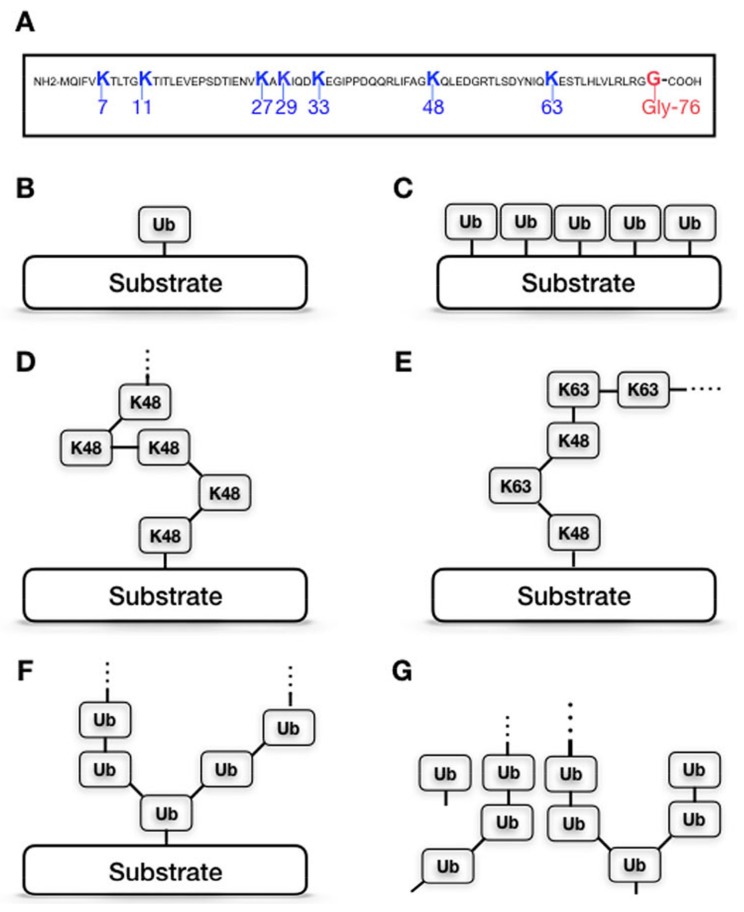
Ubiquitination is a versatile post-translational modification. Ubiquitin has seven internal lysine residues (**A**) which can be used to create different chain topologies. Substrates can be ubiquitinated on more than one residue, by different types of ubiquitination. Substrates can be mono-ubiquitinated (**B**), multimono-ubiquitinated (**C**), poly-ubiquitinated by one or more homogenous ubiquitin chain (**D**), or one or more heterogenous poly-ubiquitin chain (**E**) or by branching poly-ubiquitin chains (**F**). Unanchored Ub chains (**G**) are generated by the deubiquitination of substrates.

Ubiquitination is a fundamental mechanism regulating the half-life, localization, and activity of cellular proteins. This post-translational modification is catalyzed by the action of three types of enzymes: ubiquitin (Ub)-activating enzymes (E1s), Ub-conjugating enzymes (E2s), and Ub-ligating enzymes (E3 ligases). Their concerted activity results in a covalent attachment of ubiquitin (Ub), a 76-amino acid polypeptide, via the carboxyl group of its C-terminal glycine (Gly76) to lysine (K) residues of protein substrates. The conjugated ubiquitin can further be extended with other ubiquitin moieties via one of the internal lysine residues (K6, K11, K27, K29, K31, K48, and K63), creating polyubiquitin chains linked to the substrate [21,22,23]. Since there are seven lysine residues within the Ub polypeptide, different chain topologies, characterized by which internal lysine is linked to the next Ub moiety, can be formed (Figure 1). The linkage type of the Ub chains determines the fate of ubiquitinated substrates. The most abundant and best-characterized forms of polyubiquitination are K48- and K63-linked chains. While K48-linked chains are considered as the canonical signal for protein degradation by the 26S proteasome [24], attachment of a single Ub molecule (monoubiquitination) or K63-linked chains usually modulate the activity, localization, and intermolecular interactions of the substrate, or target it for lysosome-mediated degradation [25].

The human genome encodes for only two E1 enzymes and approximately 35 E2 enzymes [26,27]. Central to the ubiquitination process are the E3 ligases, which ultimately mediate the transfer of Ub molecules from E2s to targeted substrates [28]. E3 ligases represent by far the largest group of ubiquitin enzymes—over 600 proteins that can contribute to E3 ligase activity have been identified. The E3 ligases can generally be classified into three main groups: RING, HECT, and U-box enzymes, based on shared properties in structure and mechanism of action [29] (occasionally, the U-box enzymes are referred to as “E4” [30,31]). E3 ligases are of particular interest in regard to cancer biology, given that they confer target specificity to ubiquitin-dependent pathways, and because they are frequently deregulated in human cancer [32]. The RING-type ligases represent the most numerous group of E3 enzymes with more than 350 annotated proteins [33]. The HECT-type subgroup has approximately 30 members and the U-box-type subgroup has only 12 characterized members [34,35].

Ubiquitination is a reversible process; ubiquitin chains can be removed from ubiquitinated substrates by a special group of proteases, usually referred to as deubiquitinating enzymes (DUBs). DUBs cleave the covalent bond between the C-terminal glycine of the most proximal ubiquitin moiety and the ubiquitinated lysine of the substrate. Deubiquitination is important for cellular function for several reasons. First, DUBs are responsible for processing inactive ubiquitin precursors into active ubiquitin molecules available for covalent attachment to protein substrates. Second, they are necessary for maintaining a sufficient pool of free ubiquitin molecules within the cell to ensure a balanced rate of proteolysis. In addition, DUB enzymes are required to protect the 26S proteasome from free ubiquitin chains (Figure 1) that could compete with ubiquitinated substrates for proteasomal ubiquitin-binding sites [36]. Most importantly, however, DUBs act in concert with the ubiquitin-ligase machinery to ensure the proper steady state of ubiquitination of cellular proteins.

Approximately 100 human DUB enzymes have been identified. They are often classified, based on their protease domains, into four subgroups: ubiquitin-specific proteases (USPs), ubiquitin C-terminal hydrolases (UCH), Otubain proteases (OTU), and Machado-Joseph disease proteases (MJD). Most DUBs are cysteine proteases. However, a small number of the DUB enzymes are metalloproteases and are then often referred to as JAMMs (JAB1/ MPN/Mov34 metalloenzymes) [37].

Protein homeostasis is essential for living cells and for organisms to maintain normal cellular functions [38]. Its deregulation can lead to human disease [38,39]. Ubiquitination and deubiquitination majorly contribute to the regulation of protein degradation, which is essential for proper protein homeostasis [39]. Almost all cellular proteins are subjected to these modifications at least once in their lifetime. Thus, ubiquitination can regulate virtually all signal transduction pathways including Hippo signaling [37,40,41]. Many human diseases are strongly associated with deregulation of the ubiquitin system [42]. Recent studies have provided evidence that ubiquitinating and deubuiquitinating enzymes act to regulate turnover of multiple Hippo components and thus Hippo pathway activity. In this article, we will focus our review on ubiquitin-dependent regulation of major components of the mammalian Hippo pathway. We also briefly discuss evidence for deregulation of these enzymes in human cancer and how these enzymes could potentially be exploited for cancer therapy.

## 2. Regulation of the Hippo Pathway by Ubiquitinating Enzymes

**Figure 2:**
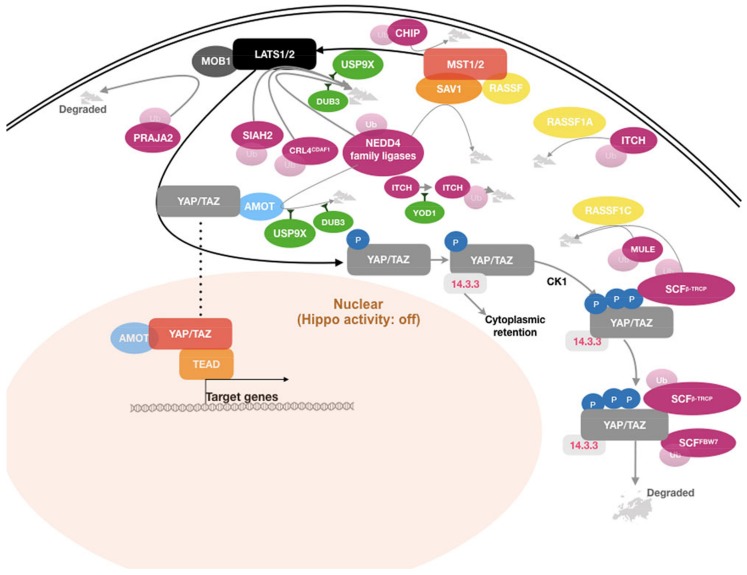
Graphic representation of key components of the Hippo signaling network, indicating major ubiquitin-dependent regulatory factors and how they impact the pathway.

Multiple nodes in the Hippo pathway have been identified as substrates of E3 ligases, as illustrated in Figure 2. In most cases, ubiquitin-dependent regulation of Hippo pathway components affects their stability and turnover, thereby affecting pathway activity.

### 2.1. Regulation of LATS Kinase Turnover

The core Hippo kinases LATS1 and LATS2 play a central role in the Hippo pathway. They directly phosphorylate YAP and TAZ, leading to their cytoplasmic retention by 14-3-3 proteins and subsequent degradation. Thereby, LATS-dependent phosphorylation results in inhibition of the activity of YAP and TAZ as transcriptional co-activators [5]; as such, the LATS kinases act as tumor suppressors. Thus, perturbations of the abundance or activity of LATS1/2 have a direct impact on the transcriptional activity of YAP and TAZ. A large number of studies provide evidence that the LATS kinases are substrates for several E3 ligases, the expression of which has been found altered in several types of human cancer. Ubiquitination by these ligases primes LATS1 and LATS2 for proteasome-mediated degradation, resulting in reduced LATS kinase abundance. As a consequence of this, YAP and TAZ remain underphosphorylated on the residues targeted by LATS and therefore translocate to the nucleus to induce YAP/TAZ target gene expression. This promotes YAP/TAZ oncogenic activity.

#### 2.1.1. NEDD4 Family E3 Ligases

HECT-type E3 ligases serve as catalytic intermediates in the ubiquitination process through their HECT domain (reviewed in [43]). Among this family of ligases, the NEDD4 subfamily has been most intensively studied for its role in regulating the Hippo pathway. The subfamily, which includes NEDD4-1, NEDD4-2, NEDL1, NEDL2, WWP1, WWP2, SMURF1, SMURF2, and ITCH, is characterized by shared functional domains [44]. All nine NEDD4 ligases have a C-terminal HECT domain and several N-terminal domains, which contribute to determining substrate specificity. Each ligase contains a calcium-dependent phospholipid binding domain (C2 domain) and two to four WW domains located toward its N-terminus. The WW domains are particularly relevant in regard to regulation of Hippo signaling. They contain two highly conserved tryptophan residues which specifically interact with their cognate proline-rich motifs, PPxY and LPxY, which are present on several members of the Hippo pathway (reviewed in [45,46]). Some NEDD4 E3 ligases, such as NEDD4-1 (NEDD4) and ITCH, have been reported to target multiple Hippo pathway components. Expression of these ligases enhanced oncogenic transformation in preclinical models and their deregulation has been implicated in human cancer [47,48,49,50,51,52,53].

The LATS kinases contain the conserved PPxY motifs (two in LATS1 and one in LATS2) that serve as specific binding sequences for WW-containing proteins. Several NEDD4 ligases have been reported to interact and regulate turnover of LATS1 and LATS2. ITCH was the first NEDD4 ligase shown to regulate Hippo signaling and was attributed a potential role in oncogenic transformation [47]. ITCH was demonstrated to physically interact with and target LATS1 for ubiquitination-dependent proteasomal degradation [47,48]. The interaction is mediated by the two PPxY motifs of LATS1 and the four WW domains of ITCH. ITCH-mediated degradation of LATS1 led to enhanced cell growth and increased tumorigenicity [47,49]. Interestingly, ITCH expression was found significantly upregulated in several cancers, such as invasive and metastatic breast cancer, pancreatic tumors, and squamous cancer of the cervix (SCC). In breast and pancreatic tumors, the expression levels of ITCH correlated with reduced cancer survival [49,50]. In SCC tumors, the increased expression of ITCH and the low level of LATS1 were strongly associated with the progression of precancerous lesions to SCC [54]. These data suggest that ITCH-Hippo signaling might be relevant to human cancer and ITCH could therefore potentially be exploited as a potential target for cancer treatment.

Several other NEDD4 E3 ligases were also reported to regulate turnover of the LATS kinases through a similar mechanism. NEDD4-1 was shown to target both LATS1 and LATS2 for ubiquitination and subsequent proteasome-mediated degradation [51,52]. WWP1 was reported to target LATS1 for degradation mediated by polyubiquitination and the 26S proteasome pathway. Degradation of LATS1 was demonstrated to be critical for the effect of WWP1 in promoting cell proliferation of breast cancer cells [53].

The evidence that NEDD4 E3 ligases are involved in carcinogenesis through promoting degradation of Hippo kinases present them as attractive molecular targets for cancer therapy. NEDD4-specific small-molecule inhibitors have been considered as potential anticancer drugs, especially in light of previously reported successful strategies targeting protein kinases. Indeed, much effort has been made to develop inhibitors for oncogenic E3 ligases [55]. Compared to the general proteasome inhibitors, some of which have been tried for cancer treatment in a clinical setting [56], Ub ligase inhibitors are expected to have fewer side effects and are anticipated to be more selective for tumor cells. The recently identified small-molecule Heclin (*N*-(4-Acetylphenyl)-3-(5-ethyl-2-furanyl)-2-propenamide) was shown to specifically target the E2 binding sites on the HECT domains of several NEDD4 family ligases such as SMURF2, NEDD4-1, and WWP1 [57]. In vitro data showed that Heclin can prevent NEDD4 ligase-mediated substrate degradation [58,59]. More data are needed to assess the effect of Heclin on growth and viability of cancer cells, especially in regard to its ability to reactivate Hippo signaling. However, it is tempting to speculate that Heclin might be a useful tool to investigate the effect of inhibiting the activity of NEDD4 ligases in relevant tumors. Molecular survey of patient tumors for deregulated E3 ligases and YAP-dependent expression signatures might be a promising way forward to identify patients that might benefit from such a targeted treatment, although tumor heterogeneity and nonexclusive target specificity of E3 ligases remain a major challenge.

#### 2.1.2. RING-Type E3 Ligases

RING-type E3 ligases function by mediating the transfer of Ub moieties directly from E2 enzymes to targeted substrates (reviewed in [28]). The RING domain of these ligases coordinates two zinc ions in a cross-braced arrangement to create a platform for E2-enzyme binding, which mediates the transfer of Ub molecules to the substrate. The substrate recognition and the mechanism of Ub transfer from E2s to substrates are diverse among the RING ligases; they can function as monomers, dimers, or can assemble into functional multi-subunit complexes. Several RING-type ligases have been studied for their role in regulating turnover of Hippo pathway components.

##### Siah E3 Ubiquitin Protein Ligase 2, SIAH2

SIAH2, a RING-type E3 ligase, is an essential component of the hypoxia-response pathway [60]. It acts by targeting prolyl-4-hydroxylase domain-containing enzymes and thereby inhibits degradation of hypoxia-inducible factor 1. SIAH2 was demonstrated to promote LATS2 ubiquitination and subsequent proteasomal degradation [61]. The interaction between SIAH2 and LATS2 was significantly augmented under hypoxia conditions. Consequently, hypoxia-induced or exogenously expressed SIAH2 inhibited LATS2 activity and YAP phosphorylation at serine S127, thus promoting transcriptional activity of YAP, cell viability and proliferation in vitro, and in xenograft tumors of MDA-MB-231 cells. Conversely, targeting hypoxia-activated SIAH2 by siRNAs restored expression and activity of LATS2, leading to growth arrest and apoptosis. Downregulated LATS2 was found in a majority of human breast tumors and, consistently, its expression negatively correlated with the expression levels of SIAH2. This may indicate that SIAH2-mediated regulation of LATS2 turnover may play an important role in this cancer. Hypoxia is a common feature of solid tumors, as is reduced Hippo signaling. The evidence that hypoxia-induced SIAH2 stimulates transcriptional activity of YAP suggests that the hypoxic microenvironment in solid tumors might contribute to support cancer cell growth by inhibiting Hippo pathway activity. In patient samples, SIAH2 has been found upregulated in breast carcinoma [61], breast ductal carcinoma [62], hepatocellular carcinoma [63], and prostate adenocarcinoma [64]. Taken together, the data discussed above suggest that SIAH2 is an interesting candidate to be explored as a therapeutic target for cancer treatment. The recently developed covalent SIAH2 inhibitors, BI-107F7 and BI-107F9 [65], which act by disrupting the interaction between SIAH2 and its substrates, might serve as chemical probes for assessing the role of SIAH2 inhibition for cancer therapy.

##### CRL4^DCAF1^

Another RING-type E3 ligase, CRL4^DCAF1^, was also reported to inhibit Hippo signaling through regulating turnover of LATS1/2 [66]. CRL4^DCAF1^ was demonstrated to interact with the LATS kinases in the nucleus, resulting in their ubiquitination and proteasome-mediated degradation. The regulation of LATS turnover by CRL4^DCAF1^ was shown to be particularly active in cells derived from tumors that either harbor inactivating mutations in the NF2 gene or that are characterized by reduced or abolished NF2 expression. NF2 is an important mediator of Hippo signaling that acts by connecting LATS kinases and their upstream activating kinases MST1/2 at the cell membrane [15]. In NF2-deficient cells, expression of CRL4^DCAF1^ enhanced oncogenic transformation. Conversely, depletion of CRL4^DCAF1^ led to increased stability of the LATS kinases and suppression of YAP activity, resulting in inhibition of cell growth and proliferation. In addition, the interaction between CRL4^DCAF1^ and LATS kinases was disrupted in the presence of active NF2, likely by competition between NF2 and CRL4^DCAF1^ for a common binding site on the LATS kinases. This is consistent with an earlier report that showed that NF2 inhibits the ligase activity of CRL4^DCAF1^ [67]. Expression analysis of YAP/TAZ target genes in different types of NF2-deficient tumors corroborated that the CRL4^DCAF1^-LATS signaling connection sustains the oncogenicity of NF2-deficient tumor cells. Given that loss-of-function mutations and reduced NF2 expression are frequently observed in human cancer [7], it is interesting to speculate that the CRL4^DCAF1^-LATS axis might be a potential target for cancer treatment, especially for a subset of patients with NF2 lesions.

### 2.2. Regulation of YAP and TAZ Turnover

Activated LATS kinases phosphorylate YAP at multiple residues, including the five LATS candidate consensus HxRxxS motifs harboring serine (S) residues S61, S109, S127, S164, and S381 [68]. There are four such consensus motifs present on TAZ [69]. While phosphorylation of YAP at S127 (corresponding to S89 on TAZ) was shown to promote its binding to 14-3-3 proteins and consequently its cytoplasmic retention [5], phosphorylation of YAP at S381 (S311 on TAZ) creates a binding site for casein kinase 1 (CK1). Subsequent phosphorylation of YAP and TAZ by CK1 at the DSGxS motif creates the canonical phosphodegron DpSGxpS, which is a specific recognition site for β-TRCP, a substrate recognition subunit of a multi-subunit SKP-CULLIN-F-box (SCF) ligase complex [68,69]. In this modular assembly, β-TRCP, a RING-type ligase and F-box motif-containing protein, confers substrate specificity, and a CULLIN serves as scaffolding protein for β-TRCP and a SKP protein. The SKP protein acts as a linker connecting β-TRCP with another RING-type ligase RBX, which mediates interaction of the SCF complex with the E2 enzyme [70,71]. A reconstituted SCF^β-TRCP^ complex containing SKP1, CULLIN1, RBX1, and β-TRCP was able to recognize and ubiquitinate TAZ in vitro [68,69]. Expression of this complex promoted proteasome-mediated degradation of YAP and TAZ in vivo [68,69]. Taken together, these results suggest β-TRCP might act as a tumor suppressor through regulating YAP and TAZ turnover.

Recently, another RING E3 ligase and F-box protein, FBW7, was shown to regulate YAP abundance in hepatocellular carcinoma (HCC) cell lines [72]. Knockdown of FBW7 resulted in increased YAP stability, while its overexpression led to YAP ubiquitination and degradation in HepG2 and Hep3B cells. Consistent with its activity in regulating YAP turnover, FBW7 expression was also demonstrated to inhibit cell proliferation and induce apoptosis. FBW7 is often expressed at low levels in HCC tumors, as compared to adjacent normal tissue [72]. In addition, FBW7 expression was found inversely correlated with YAP expression in human HCC samples, and strongly associated with better cancer survival of HCC [72] and intrahepatic cholangiocarcinoma [73]. Moreover, low FBW7 expression level was identified as an independent prognostic factor of liver cancer recurrence after surgery [74]. These data suggest that FBW7 is a potential tumor suppressor, exerting its growth-suppressive effect at least in part through regulating turnover of YAP, especially in the context of HCC. So far, an impact of the FBW7-YAP axis has not been reported in other cancers than that of the liver. However, FBW7 is known to also target several other oncogenic targets, such as MYC, JUN, and Cyclin E [75]. Loss of FBW7 expression also leads to chromosomal instability, likely a consequence of its hyperactivated oncogenic substrates. Moreover, mutations of FBW7 have been found in diverse human tumors (reviewed in [75,76]). The identification of YAP as another oncogenic target of FBW7 further validates it as an important tumor suppressor.

### 2.3. Regulation of Angiomotin Turnover

The tight junction angiomotin (AMOT) proteins have been shown to stably associate with several components of the Hippo pathway, and these interactions have been implicated in regulating the activity of YAP and TAZ [77,78,79,80]. AMOT proteins interact with YAP and TAZ through the WW domain-L/PPxY motif interaction independent of their phosphorylation status. This interaction results in an inhibitory effect on transcriptional activity of the Hippo effectors. Moreover, LATS1/2 bind and phosphorylate AMOT at the serine residue within the conserved HxRxxS motif, enhancing its stability and its inhibitory effect on YAP [81,82,83,84]. In addition, AMOT proteins interact with NF2 through their coiled-coil domains [85,86]. The association induces the functionally active conformation of NF2 as well as its interaction with LATS kinases, promoting transduction of the Hippo signaling pathway to inhibit YAP and TAZ activity [15,86]. The PPxY-containing p130/AMOT also interacts with WW domain-containing KIBRA, an upstream regulator of the Hippo pathway [87]. However, the effect of the KIBRA-p130/AMOT interaction on Hippo activity is difficult to assess directly, given that KIBRA also interacts with LATS kinases and is involved in regulation of apical-basal cell polarity, both of which in turn can affect Hippo signaling activity [88,89,90]. Additionally, p130/AMOT was also proposed to play a scaffolding role in mediating the inhibitory effect of ITCH on YAP, resulting in ubiquitination and subsequent degradation of YAP [91,92]. AMOTL2 was also found to bind to MST2, though the domains mediating this interaction were not characterized [93]. AMOTL2 was suggested to act through this interaction as a scaffolding protein, promoting the activity of the LATS kinases on YAP. Taken together, these studies demonstrated that AMOT proteins contribute to suppress the transcriptional activity of YAP and TAZ. Two recent studies suggested that p130/AMOT might be required for YAP function in certain cells lacking NF2 expression, which is required for correct localization and functions of LATS2 on YAP [15]. In NF2-null mouse hepatocytes and in NF2-null Schwann cells, enhanced binding between hyperphosphorylated p130/AMOT and LATS kinases was shown to interfere with the LATS-YAP interaction, preventing LATS-dependent phosphorylation of YAP; unphosphorylated p130/AMOT, however, localized to the nucleus, where it facilitated the interaction between YAP and TEAD [83,94]. Thus, AMOT might mediate a positive effect on transcriptional activity of YAP in particular cellular contexts.

Several NEDD4 family E3 ligases have been reported to interact with and regulate the abundance and stability of the AMOT proteins. Three of these WW-containing ligases, NEDD4-1, NEDD4-2, and ITCH, were shown to interact with p130/AMOT though its L/PPxY motif, promoting its cytoplasmic translocation, ubiquitination, and proteasomal degradation. Conversely, inhibition of these E3 ligases stabilized p130/AMOT [95]. These data suggest that in addition to degrading the LATS kinases, the NEDD4 ligases act to suppress YAP activity through regulating turnover of AMOT proteins. Interestingly, the interaction between the NEDD4 ligases and AMOT appears to be mediated by the same WW-PPxY interacting module as that of YAP and AMOT, respectively, raising a possibility that YAP, which has not been characterized as a NEDD4 substrate, might compete with the NEDD4 ligases for binding with the AMOT proteins. If so, it remains to be determined if high expression levels of YAP protect the AMOT proteins from degradation mediated by the E3 ligases. In addition, the NEDD4 ligases were also reported to have differential effects on the stability of AMOT proteins under some experimental conditions. For example, NEDD4L2 was reported to enhance stability of AMOTL1 via K63-linked polyubiquitination in endothelial cells [92]. Another study showed that ITCH stabilized p130/AMOT in HEK293T and MDA-468 cells [91], though the mechanism remains unclear. These results suggest that AMOT-targeting E3 ligases might have differential effect on Hippo signaling, depending on the cellular context.

### 2.4. Regulation of MOB1 Turnover

MOB1 proteins, encoded by the paralogous genes MOB1A and MOB1B, are key regulators of LATS1/2 activity. Activation of the LATS kinases requires a sequential and multistep process involving the MST kinases and MOB1. First, the MST kinases undergo activating autophosphorylation [96]. Autophosphorylation occurs at multiple serine and threonine residues between the kinase and SARAH domains, creating a docking site for MOB1 binding. This interaction catalyzes MOB1 phosphorylation by the MST kinases. Phosphorylated MOB1 undergoes an activating conformational change, which allows MOB1 to act as an adapter between MST and LATS kinases. This enables the phosphorylation of the LATS kinases by the MST kinases [97,98,99,100]. PRAJA2, a RING-type E3 ligase, was recently shown to bind to MOB1, ubiquitinate, and thereby target it for proteasomal degradation. Consequently, PRAJA2 activity reduced Hippo signaling [101]. Consistently, ectopic expression of PRAJA2 led to a stimulation of transcriptional activity of YAP. Interestingly, PRAJA2 has been found frequently overexpressed in glioma tumors [101]. Its overexpression was accompanied by a suppression of MOB1 expression and increased nuclear localization of YAP [101], suggesting that PRAJA2 may play a pivotal role in tumorigenesis of glial cells. Another recent study reported that PRAJA2 is overexpressed in thyroid tumors, though Hippo signaling was not investigated in this study [102]. These studies suggest that PRAJA2 might be a novel therapeutic target for some types of cancer.

### 2.5. Regulation of MST1/2 Turnover

MST kinases play a central role in the Hippo pathway by directly phosphorylating and activating the LATS kinases. Activation of MST kinases can be achieved by TAOK1, 2, 3 kinases, which phosphorylate the MST activation loop (Thr183 of MST1 or Thr180 of MST2) [103,104]. Additionally, there is evidence suggesting that MST1/2 can be activated by dimerization, which is mediated through their Salvador-RASSF-Hippo (SARAH) domains, and autophosphorylation of the activation loop [105,106]. Stability and phosphorylation status of the MST kinases are critical to downstream Hippo pathway signaling.

Recently, the U-box family protein E3 ligase CHIP was reported to promote proteasomal degradation of MST1/2 [107,108]. MST abundance was demonstrated to be strongly influenced by the expression level of heat shock protein 70 (HSP70). HSP70 interacts and recruits MST1/2 to its interacting partner, CHIP, resulting in ubiquitination and subsequent degradation of the MST kinases. Currently, little is known about CHIP expression in human cancers. However, HSP70 has been found overexpressed in most human cancers compared to adjacent normal tissue and it is considered a molecular target for cancer treatment (reviewed in [109]). These data might imply HSP70/CHIP as a negative regulator of Hippo pathway signaling and the HSP70/CHIP-MST regulatory axis may be explored for cancer treatment.

### 2.6. Regulation of SAV1 Turnover

The highly conserved SAV1 protein, also referred to as WW45, has been identified as a tumor suppressor in human cancer, acting as a cofactor/adaptor/regulator of the MST kinases and promoting Hippo signaling [110,111,112]. SAV1 can form a heterodimer or heterotetramer with MST kinases through their SARAH domains [113,114]. The SAV1-MST interaction was demonstrated to be required for the correct cellular localization, activation, and function of the MST kinases [114,115]. Additionally, SAV1 was shown to maintain MST activity by antagonizing the PP2A-containing phosphatase complex [114]. SAV1 also interacts with other PPxY motif-containing Hippo components, such as LATS kinases and AMOT proteins, through its WW domain [113]. The LATS-SAV1 interaction recruits LATS to the MST, resulting in LATS phosphorylation by MST1/2 [116,117]. However, the biological effect of the AMOT-SAV1 association remains to be determined. In human 293T cells, the NEDD4-1 E3 ligase was shown to bind and ubiquitinate SAV1, promoting its degradation [52]. Given the NEDD4 functions described above, this further corroborates the central function of this E3 ligase as a key regulator of Hippo signaling.

### 2.7. Regulation of RASSF Turnover

In addition to SAV1, several RAS-associated domain (RA) family (RASSF) proteins have been shown to act as cofactors of the MST kinases, thereby regulating Hippo pathway activity. The RASSF family represents a group of 10 adaptor proteins that share a RA domain at their N-termini, but diverge in whether they contain (RASSF1-6) or do not contain (RASSF7-10), a SARAH domain at their C-termini [118,119]. RASSFs behave as tumor suppressors—their depletion, loss of heterozygosity, or epigenetic silencing is frequently observed in human tumors. Conversely, their overexpression results in proapoptotic or antiproliferative effects in cancer cell lines (reviewed in [120]). RASSF1A is the best characterized member and high expression of the gene has been found to positively correlate with better prognosis of many human solid cancers (reviewed in [121,122]). The RASSF proteins act primarily by mediating protein–protein interactions critical for signal transduction, including in the RAS and Hippo pathways. While the presence of RA domains, which interact with activated GTP-RAS proteins, may suggest that RASSFs are effectors of activated RAS, the SARAH domain links RASSF proteins to the Hippo pathway. RASSF1A is perhaps the best understood example of a RASSF protein regulating Hippo pathway activity. The SARAH domains of RASSF1A and the MST kinases interact and thereby promote heterodimerization [113,123,124]. This leads to enhanced autophosphorylation and activation of the Hippo kinases [125,126,127,128,129,130,131] by preventing their dephosphorylation [132]. Other structurally related members such as RASSF2, RASSF4, and RASSF5 have also been found to activate MST kinases in a similar manner [131,133,134,135,136].

Several E3 ligases have been shown to ubiquitinate RASSF proteins to target them for proteasomal degradation. In U2OS and H1299 cells, TGFβ treatment resulted in RASSF1 recruitment to TGFβ1R and ITCH, which resulted in RASSF1 ubiquitination by the E3 ligase [137]. ITCH-mediated degradation of RASSF1A was necessary to permit YAP1’s association with the transcription factor SMAD2, which subsequently promoted transcriptional activation of TGFβ-responsive target genes. ITCH was also demonstrated to ubiquitinate RASSF5, which is closely related to RASSF1A, and target it for degradation both in vivo and in vitro [138]. The interaction between ITCH and RASSF5 is mediated through the canonical WW domain-PPxY motif. Another study provided evidence that SKP2, a subunit of the SCF ligase complex, interacts with, mediates ubiquitinaton of, and thereby promotes degradation of RASSF1A during the G1-S transition of the cell cycle [139]. A less stable isoform of RASSF1A, RASSF1C was shown to be targeted by MULE (a HECT-type E3 ligase) under normal conditions or both MULE and SCF ^β-TRCP^ in response to UV irradiation [140]. These data indicate that E3 ligases such ITCH and β-TRCP can influence Hippo pathway signaling by regulating the turnover of MST1/2 regulatory RASSF proteins.

## 3. Regulation of Hippo Signaling by Deubiquinating Enzymes

The activity of ubiquitin ligases on target cellular proteins is counteracted by DUBs, which catalyze the removal of covalently attached ubiquitin moieties. The balance between the ubiquitination and deubiquitination processes is critical for maintaining proper protein homeostasis, which is essential for normal cell viability and functions. We previously performed a shRNA-based screen and identified several DUBs, the knockdown of which affected YAP/TAZ activity [141]. In a recent study, a similar screen using siRNAs found additional DUBs whose inhibition regulated the protein levels of YAP and TAZ [142]. Several Hippo components have been identified as targets of DUB enzymes and the expression of these DUBs has been found altered in some human cancers.

USP9X, a DUB that belongs to the USP subclass, has been identified by several independent groups to positively regulate Hippo pathway activity [141,142,143,144]. USP9X were reported to interact with several Hippo proteins, including LATS1/2, AMOT, SAV1, and KIBRA [141,143]. Catalytically active USP9X was shown to remove Ub molecules attached to K496 of AMOT, leading to its enhanced stability and suppression of YAP transcriptional activity [141]. Mutation of this lysine residue significantly abolished the stabilized effect of USP9X on AMOT, indicating that ubiquitination at K496 residue is a major mechanism for Ub-mediated proteasomal degradation of AMOT. In addition, USP9X was also demonstrated to stabilize LATS kinases [143,144], resulting in a suppression of YAP activity. Generally, depletion of USP9X led to reduced expression of its Hippo pathway substrates and consistently increased expression of known YAP targets. Taken together, these observations suggest that USP9X acts to promote stability of several Hippo pathway components, therefore inhibiting oncogenic activity of YAP. USP9X expression has been found to be reduced in several types of human cancer, such as pancreatic [143,145], and in kidney tumors [141] and breast cancer cell lines [143]. Furthermore, reduced USP9X expression was found to be associated with poor survival in colorectal cancers [146]. These data suggest that USP9X might act as a tumor suppressor in these cancers. Interestingly, USP9X has been found overexpressed in some other human cancers, such as non-small cell lung cancer, multiple myeloma, and squamous cell carcinoma [147,148,149,150], implying that in different contexts, it also may act as an oncogene. The broad substrate specificity characteristic for DUBs is the most likely explanation for this apparent contradiction. For example, the antiapoptotic proteins MCL1 and SURVIVIN were shown to be stabilized by USP9X, promoting oncogenic transformation [150,151].

Several other DUBs have also been reported to act as tumor suppressors through regulating the Hippo pathway. USP21 was shown to interact and promote the stability of MARK proteins, which are upstream regulators of the Hippo pathway. Consequently, inhibition of USP21 led to a suppression of LATS activity and increased nuclear YAP localization. USP21 protein was found to be expressed at low levels in a majority of human kidney clear cell tumors [152]. Yet another DUB implicated in regulating the Hippo pathway is DUB3. The cellular proteins which DUB3 targets to regulate Hippo signaling remain unknown. However, DUB3 knockdown was found to suppress LATS phosphorylation and to increase nuclear localization of YAP [153]. Of note, DUB3 was reported to influence cancer-relevant pathways by additional means [154,155].

YOD1, which belongs to the OTU subclass of DUB enzymes, was found to interact with and regulate the stability of the ITCH E3 ligase. YOD1-mediated stabilization resulted in degradation of the LATS kinases, consequently leading to an increased transcriptional activity of YAP and TAZ [142]. Interestingly, inducible expression of YOD1 in the mouse liver caused hepatomegaly, a phenotype resembling that of YAP overexpression. Furthermore, YOD1 was found upregulated in 60% of human hepatocellular carcinoma tumors examined, as compared to adjacent normal tissue. YOD1 expression levels correlated strongly with nuclear YAP staining. These results suggest that elevated YOD1 levels or activity may play an oncogenic role in liver cancer through suppressing Hippo activity. Thus, YOD1 inhibitors may potentially be assessed as a therapeutic tool to reactivate Hippo signaling in liver cancer.

## 4. Concluding Remarks

The above described data suggest that ubiquitination machinery plays an essential role in regulating the Hippo pathway. Ubiquitinating and deubiquinating enzymes can act at multiple nodes in the Hippo signaling pathway to regulate its activity (Figure 2). The abundance of Hippo core components including the LATS kinases, MSTs, AMOT, YAP, and TAZ has been shown to be strongly influenced by the ubiquitin system. Most notable are, perhaps, independent reports that show that LATS1 and 2 are substrates of several E3 ligases and DUBs. The multiple ubiquitin enzymes act, possibly in a (maybe partially) redundant manner or dependent on the specific cellular context, to regulate turnover of the core Hippo kinases, indicating the abundance and activity of the LATS kinases must be under a tight control. It should be noted that the Hippo pathway can be regulated by a number of different upstream signals, including G-protein coupled receptor (GPCR) signaling, CRB, KIBRA, and NF2 [3]. Not unexpectedly, these regulators are also regulated by the ubiquitin machinery, arguing for additional levels of Ub-dependent Hippo pathway activity regulation.

Stable and specific associations between WW domain- and proline-rich (PPxY/F) motif-containing proteins have been demonstrated to be critical to the control of the modular cascade of the Hippo pathway (reviewed in [46]). The signal transduction between LATS1/2 and their effectors YAP and TAZ is centrally mediated through the WW domain (YAP/TAZ)—PpxY motif (LATS1/2) interaction [156,157]. Mutations in the WW domains or the PPxY motifs were demonstrated to effectively disrupt binding between LATS kinases and their effectors [157,158]. Many other important physical interactions in the pathway, for example those between AMOT-YAP/TAZ and SAV1-LATS, are mediated through these domains. It is noteworthy that an unusually high number of core Hippo components and regulators are either WW-containing proteins (YAP, TAZ, SAV1, KIBRA), PPxY (LATS1/2, AMOT proteins, CRB, RASSF5), or PPxF (MST1/2) containing proteins [46,159]. Importantly, the WW-PPxY interaction module also appears to mediate Ub-dependent degradation of Hippo components, indicating that competition between E3 ligases and modulators of the pathway may play a role in ultimately regulating YAP/TAZ activity. Several members of the NEDD4 family E3 ligases, which contain several WW domains, have been demonstrated to bind and target PPxY-containing Hippo proteins for ubiquitination, as discussed above. For example, the direct and specific association between the WW domains of ITCH and PPxY of LATS1 was shown to mediate ubiquitination and therefore degradation of LATS1 both in vitro and in vivo [47,48], highlighting the biological importance of Hippo protein-E3 ligase interactions. The number of the relevant interaction domains present in the competing proteins may have some impact on the strength of interaction between partner proteins. A recent study supports that possibility. ITCH, with four WW domains, was demonstrated to compete with YAP, which has only two WW domains, for binding to p73, another PPxY-containing substrate of ITCH [158]. However, as of now, there is little evidence as to how the WW-PPxY interactions are regulated under physiological and disease conditions, and how they respond to changes of cellular condition and context. For example, ITCH has higher binding affinity to LATS1 (and consequently is more efficient to ubiquinate LATS1) under low YAP activity conditions (high density and MST2 expression) [47,48]. These data suggest that the E3 ligase might contribute to maintaining the fine balance between cell death and cell survival.

Deregulation of Hippo regulatory networks, including the ubiquitin enzymes, might be explored for cancer therapy. Although Hippo pathway activity is frequently suppressed, and the activity or expression of YAP and TAZ is elevated in cancer, it is important to note that the components of the central Hippo pathway cascade, including LATS1, LATS2, YAP, and TAZ, are not frequently mutated or deleted. This is in contrast to other major tumor suppressors, such as p53 and p16INKA/Rb, whose activity is often lost in tumor cells due to deletion or mutations [160]. Suppressed Hippo pathway signaling or increased YAP activity is likely a consequence of abnormal expression of upstream regulators, for example, overexpression of several NEDD4 ligases observed in human tumors. This raises a possibility that the suppressed Hippo pathway may be amendable for cancer therapy purposes. Deregulation of the signaling networks or regulators that lead to suppression of the Hippo pathway might be targeted to reactivate Hippo activity in tumor cells.

Recent advances in gene expression technologies have enabled tremendous progress in the understanding of cancer biology and development of targeted therapy approaches. Genome wide expression information of tumor samples can be performed relatively easily or can be obtained from accessible web portals such as cBioportal.org and EMBL-EBI. These data might permit a thorough investigation of differential gene expression between tumor and adjacent normal tissues to identity any deregulated genes in cancer, including those that regulate the Hippo pathway. A number of the ubiquitin-modifying enzymes have been found abnormally expressed through this approach and were considered as targets for cancer treatment [32,161,162]. Currently, very few studies were dedicated to investigating whether these deregulated proteins contribute to tumorigenesis through suppressing Hippo pathway signaling. It might be possible to identify a subset of patients who might be responsive to targeting of deregulated nodes of the Hippo pathway via ubiquitin enzymes. For example, identification of a significant association between upregulation of Hippo-regulating E3 ligases and the elevated expression of YAP transcriptional targets in particular types or sets of tumors might suggest the involvement of that E3 enzyme in cancer formation through inhibiting the Hippo pathway. Thus, the deregulated enzymes might be explored as molecular targets for cancer therapy for those patients. Of note, E3 ligases rarely target only a single substrate. Thus, it will be crucial to unravel their specificity spectrum in order to predict whether a given E3 ligase can be productively targeted without unwarranted side effects.

In this article, we largely limit our review on the Ub-mediated modification that regulates the stability of mammalian Hippo components and their implications in cancer. It is noteworthy, however, that other types of lysine modifications, such as sumoylation, acetylation, and methylation, which potentially compete with ubiquitination for lysine residues [163], have been reported to regulate stability or activity of Hippo proteins. For example, sumoylation of YAP induced by PML was demonstrated to prevent its ubiquitination and subsequent proteasome degradation [164]. Acetylation of YAP, which occurred upon treatment with *S*N2 alkylating agents, was found essential for its transcriptional activity [165]. The SET domain-containing lysine methyltransferase 7 (SET7) was shown to induce YAP monomethylation, which was found to be critical for its cytoplasmic retention [166]. Currently, available data of these lysine modifications of YAP and other Hippo components remain limited. Further investigation might help to better understand these modifications that contribute to the regulation of the tumor-suppressor Hippo pathway in normal and cancer cells, opening new avenues for targeting the Hippo pathway for cancer treatment.
